# Impact of Parameterization of Physical Processes on Simulation of Track and Intensity of Tropical Cyclone Nargis (2008) with WRF-NMM Model

**DOI:** 10.1100/2012/671437

**Published:** 2012-06-04

**Authors:** Sujata Pattanayak, U. C. Mohanty, Krishna K. Osuri

**Affiliations:** Centre for Atmospheric Sciences, Indian Institute of Technology, Delhi, Hauz Khas, New Delhi 110016, India

## Abstract

The present study is carried out to investigate the performance of different cumulus convection, planetary boundary layer, land surface processes, and microphysics parameterization schemes in the simulation of a very severe cyclonic storm (VSCS) Nargis (2008), developed in the central Bay of Bengal on 27 April 2008. For this purpose, the nonhydrostatic mesoscale model (NMM) dynamic core of weather research and forecasting (WRF) system is used. Model-simulated track positions and intensity in terms of minimum central mean sea level pressure (MSLP), maximum surface wind (10 m), and precipitation are verified with observations as provided by the India Meteorological Department (IMD) and Tropical Rainfall Measurement Mission (TRMM). The estimated optimum combination is reinvestigated with six different initial conditions of the same case to have better conclusion on the performance of WRF-NMM. A few more diagnostic fields like vertical velocity, vorticity, and heat fluxes are also evaluated. The results indicate that cumulus convection play an important role in the movement of the cyclone, and PBL has a crucial role in the intensification of the storm. The combination of Simplified Arakawa Schubert (SAS) convection, Yonsei University (YSU) PBL, NMM land surface, and Ferrier microphysics parameterization schemes in WRF-NMM give better track and intensity forecast with minimum vector displacement error.

## 1. Introduction

Tropical cyclones are serious threats to human life and property. Even with the recent rapid improvements in numerical weather prediction, tropical cyclone forecasting remains a challenging problem to atmospheric modeling groups. The Bay of Bengal is a potentially active region for development of cyclonic storms and an average of five tropical cyclones annually forms over the region, representing 5.85% of the global frequency [1]. Moreover, the Bay of Bengal storms are exceptionally devastating, especially when they make landfall [2]. This is mainly due to a densely populated and low lying coastline. With the Bay of Bengal tropical cyclones being the deadliest natural disasters in the Indian subcontinent, it significantly impacts the socioeconomic conditions of the countries bordering the east coast of India.

Cumulus convection, surface fluxes of heat, moisture, and momentum and vertical mixing in the PBL play important roles in the development of tropical cyclones [3]. Convection has long been recognized as a process of central importance in the development of cyclonic storms. The scales of convective clouds are too small to be resolved by the numerical models and hence need to be parameterized in terms of variables defined at each grid point. A number of parameterization schemes have been developed over the years but each have their respective limitations [4, 5]. Performance of a numerical model in tropical cyclone forecasts depends on how well the convection is parameterized in the model [6, 7]. These studies have led to an increased understanding of the importance of the boundary layer and convective processes in the tropical cyclone development. An extensive study is carried out on the impact of the parameterization of physical processes in the simulation of two severe cyclonic storms developed over the Bay of Bengal using the MM5 model [7]. This study with MM5 model suggested that the combination of MRF and Grell (or Betts-Miller) for PBL and convection schemes, respectively, consistently give better results than the other combinations. The sensitivity experiments of convection, boundary layer, and moisture processes using the MM5 model for the prediction of the Orissa Super Cyclone 1999 is carried out by Bhaskar Rao and Hari Prasad [8] and the study suggested that convective processes plays an important role in the cyclone track prediction while the PBL controls intensification. A comparison study of four PBL parameterization schemes in simulation of Hurricane Bob (1991) is presented [9] using the MM5 model. This study suggested that significant sensitivity is seen in the central pressure and maximum surface wind (10 m). The precipitation forecast in hurricanes can be just as sensitive to the formulation of the different PBL schemes. The customization of Advanced Research WRF (WRF-ARW) model is also carried out [10] for the tropical cyclones over North Indian Ocean which suggested that the combination of KF cumulus convection scheme along with the YSU PBL is providing better track and intensity forecast. All the above-mentioned studies clearly demonstrate the impact of the parameterization of physical processes in different fields of studies using the MM5 and WRF-ARW models. So, there is a need to investigate the impact of the parameterization of physical processes in simulation of tropical cyclones over the Bay of Bengal using NMM dynamic core of the WRF (WRF-NMM) model.

In the present study, NCEP mesoscale model WRF-NMM is used to simulate a very severe cyclone Nargis with sensitivity experiments carried out to explore the impact of physical parameterizations on track and intensity prediction. The sensitivity of the model simulations to initial conditions is also explored using the optimum combination of physical parameterizations.

A brief description of the model as well as the parameterization schemes used in the study is presented in Section 2. The synoptic situation for the above-mentioned cyclone used in the present study is described in Section 3. Various numerical experiments and data used are described in Section 4. Model simulated results along with the evaluation of performance of the model with different initial conditions are presented in Section 5 with the conclusions in Section 6.

## 2. Model Description

The WRF-NMM version 3.0.1 developed by National Center for Environmental Prediction (NCEP)/National Oceanic and Atmospheric Administration (NOAA) is designed to be a flexible, state-of-the-art mesoscale modeling system. It is a fully compressible, nonhydrostatic model with a hydrostatic option [11–13]. Its vertical coordinate is a hybrid sigma-pressure coordinate. The grid staggering is the Arakawa E-grid. The dynamics conserve a number of first- and second-order quantities including energy and enstrophy [14]. Forward-backward time integration scheme is used for the horizontally propagating fast waves and implicit scheme is used for the vertically propagating sound waves. Adams-Bashforth scheme for horizontal advection and Crank-Nicholson scheme for vertical advection are used by the model. The same time step is used for all terms. The Geophysical Fluid Dynamic Laboratory (GFDL) long-wave and short-wave radiation schemes are incorporated in the model. Additionally, to represent deep, moist convection in the model, various parameterizations schemes are included. All the schemes considered for this study are reasonably independent and hence useful for sensitivity experiments.

### 2.1. Planetary Boundary Layer Schemes

The Yonsei University (YSU) PBL scheme is a revised vertical diffusion package with a nonlocal turbulent mixing coefficient in the boundary layer. The major ingredient of the revision is the inclusion of an explicit treatment of entrainment processes at the top of the PBL. The YSU PBL increases boundary layer mixing in the thermally induced free convection regime and decreases it in the mechanically induced forced convection regime, which alleviates the well-known problems in the Medium-Range Forecast (MRF) PBL. Excessive mixing in the mixed layer in the presence of strong winds is resolved. Overly rapid growth of the PBL in the case of the Hong and Pan (1996) [15] is also rectified. Consequently, the YSU scheme does a better job in reproducing the convective inhibition.

The Mellor Yamada Janjic (MYJ) PBL scheme is a one-dimensional prognostic turbulent kinetic energy scheme with local vertical mixing [12, 13]. An advantage of the MYJ scheme is that it allows advection of turbulent regions during the forecast. The top of the layer depends on the TKE as well as the buoyancy and shear of the driving flow.

The NCEP Global Forecast System (NCEP GFS) PBL scheme is a nonlocal vertical diffusion scheme [16] and further described in Hong and Pan (1996). The PBL height is determined using an iterative bulk-Richardson approach working from the ground upward whereupon the profile of the diffusivity coefficient is specified as a cubic function of the PBL height. Coefficient values are obtained by matching the surface layer fluxes. A counter-gradient flux parameterization is included.

### 2.2. Cumulus Parameterization Schemes

 The Simplified Arakawa Schubert (SAS) scheme is based on Arakawa and Schubert (1974) [17] as simplified by Grell (1993) [18] and with a saturated downdraft. The major modification is done in entrainment relation to avoid the costly calculation that is necessary to find the entrainment parameter of cloud detraining at the model levels. It is very simplistic and computationally highly efficient convective parameterization scheme leads to a very realistic simulation of the mesoscale convective systems. The scheme uses a stability closure, assumes a large cloud size, parameterizes moist downdrafts, and does not assume unrealistically large lateral mixing to simulate penetrative convection [19].

 The Kain-Fristch (KF) is a deep and shallow subgrid scheme using a mass-flux approach with downdrafts [20, 21]. Mixing is allowed at all vertical levels through entrainment and detrainment. This scheme removes convective available potential energy (CAPE) through vertical reorganization of mass at each grid point. The scheme consists of a convective trigger function (based on grid-resolved vertical velocity), a mass flux formulation, and closure assumptions. 

 The Betts Miller and Janjic (BMJ) scheme is an adjustment-type scheme for deep and shallow convection relaxing towards reference profile of temperature and specific humidity determined from thermodynamic considerations [22, 23]. The scheme's structure favors activation in cases with substantial amounts of moisture in low and mid-levels and positive CAPE. The representation is accomplished by constraining the temperature and moisture fields by the convective cloud field.

 Grell-Devenyi (GD) scheme is a multiclosure, multiparameter ensemble method. It is an ensemble average of typically more than 100 types of clouds, which includes different closures such as CAPE removal, quasiequilibrium and moisture convergence, and variants of cumulus parameterization such as changes in the parameters for entrainment, cloud radius, maximum cap, and precipitation efficiency.

The detailes of the model specifications used for the present study are presented in Table 1. 

## 3. Synoptic Situation of Tropical Cyclone Nargis

Nargis was a category 4 tropical cyclone that caused worst natural disaster in the recorded history of Mynmar. In the last week of April 2008, an area of deep convection persisted near a low level circulation in the Bay of Bengal about 1150 km east-southeast of Chennai. With good outflow and low vertical wind shear, the system slowly organized into a depression at 0300 UTC 27 April 2008. The system intensified into deep depression stage at 1200 UTC 27April 2008 with a minimum central MSLP of 998 hPa and the maximum sustained surface winds of 30 kts. After 12 hours at 0000 UTC 28 April 2008, the system intensified into cyclonic storm with a minimum central MSLP of 994 hPa and the maximum sustained surface winds of 35 kts. The system further intensified into severe cyclonic storm with a minimum central MSLP of 986 hPa and the maximum sustained surface winds of 55 kts at 0900 UTC 28 April 2008 and moved in northward direction. Then around 0300 UTC 29 April 2008, the system became a very severe cyclonic storm (VSCS) with a minimum central MSLP of 980 hPa and the maximum sustained surface winds of 65 kts. The storm remained in VSCS for a period of 93 hours, that is, up to 0000 UTC 03 May 2008. The observed minimum central pressure was 962 hPa with the pressure drop of 40 hPa and the maximum sustained surface winds of 90 kts. The storm crossed southwest coast of Myanmar around 1200 UTC 02 May 2008. The system remained on the land for further 24 hours and caused extensive devastation to coastal areas.

## 4. Numerical Experiments and Data Used

The mesoscale model WRF-NMM described in section 2 is integrated up to 123 hours in a single domain with the horizontal resolution of 9 km. The model has 51 levels up to a height of 30 km in the vertical. A number of numerical experiments producing 123 hours forecasts (for each experiment) are carried out with the possible combination of four cumulus convection schemes, two PBL schemes, four land surface physics schemes and four microphysics schemes. The four convection schemes are Kain-Fritsch [20, 21, 24], Betts-Miller-Janjic [25], Grell-Devenyi [26] and Simplified Arakawa-Schubert [17–19], which thereafter referred as K, B, G and S respectively. The two PBL schemes are NCEP Global Forecast System [15, 16] and Yonsei University [27], which thereafter referred as NC and Y respectively. The four land surface physics schemes are NMM [28], Thermal Diffusion [27], Noah [29] and RUC [30, 31], which thereafter referred as N, T, NO and R respectively. The four microphysics schemes are Ferrier (New ETA) [32], WRF Single-Movement (WSM) 5-class [33, 34], WSM 6-class graupel [33, 35, 36] and Thompson et al. [37], which thereafter referred as F, W5, W6 and T respectively. The experiments are categorized into two main groups, choosing different parameterization schemes of convection, PBL, land surface and microphysics for the best possible combination. Then in the second group, the best two combinations are re-investigated with 5 additional sets of initial conditions of the same cyclone case yielding 6 groups of simulation results. Results obtained from all possible experiments are examined by comparing with the verification analysis and observations to find the best combinations towards forecasting the track and intensity of the above mentioned cyclone.

The initial and lateral boundary conditions to a limited area model are usually provided from the large scale analysis of different NWP centers in the world. The NCEP/GFS analysis and forecasts (1° × 1° horizontal resolution) have been used to provide the initial and lateral boundary conditions to the model. The TC Nargis was intensified into cyclonic storm at about 0000 UTC 28 April 2008 and hence chosen as the initial time for the model simulations. Furthermore, the evaluation of performance of the model is carried out with the results obtained from model integration at different initial conditions. For this purpose, six simulations have been carried out from the initial conditions of 0000 UTC 28 April 2008, 1200 UTC 28 April 2008, 0000 UTC 29 April 2008, 1200 UTC 29 April 2008, 0000 UTC 30 April 2008 and 1200 UTC 30 April 2008 with the optimum model configuration. Also, each simulation is done up to 0300 UTC 03 May 2008.

## 5. Results and Discussions

 The results as obtained with different combinations of parameterization schemes producing 123 hours forecasts for Nargis (as described above) are presented in this section to examine the performance of the parameterization of physical processes in the prediction of track and intensity of the tropical cyclone.

### 5.1. Sensitivity Experiments with Convection Schemes

**Figure 1:**
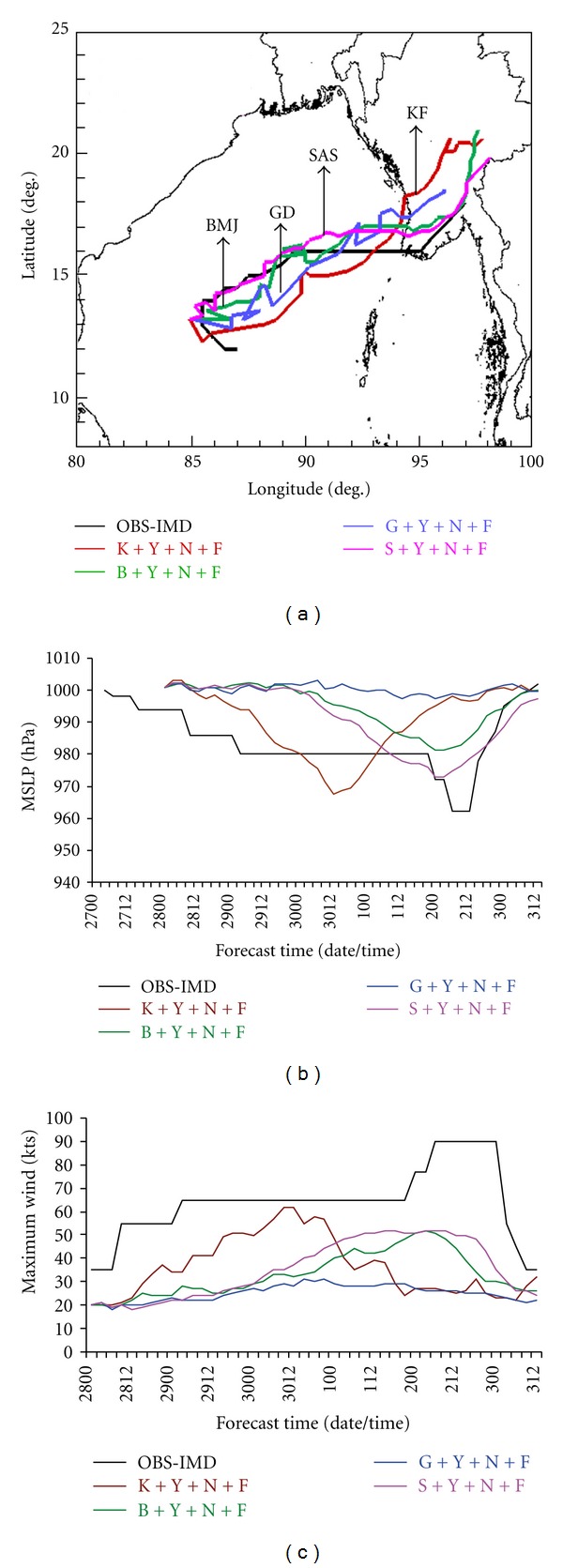
(a) Track of the cyclone Nargis, (b) MSLP (hPa), and (c) maximum wind (kts) with 4 different cumulus convective schemes, YSU PBL, NMM land surface, and Ferrier microphysics option.

 In this subsection, four experiments are carried out with the variation of the parameterization scheme for convection as Kain-Fritsch (K), Betts-Miller-Janjic (B), Grell-Devenyi (G), and Simplified Arakawa-Schubert (S) in combination with YSU (Y) scheme for PBL, NMM (N) scheme for land surface and Ferrier (F) microphysics scheme. Model-simulated track positions are presented in Figure 1(a) to facilitate the evaluation. The results indicate that B and S schemes are producing similar type of results in terms of track prediction. The movement of the cyclone with K scheme is much faster than any other schemes. The G scheme is providing the reasonable prediction of the track position. The results indicate that the movement of the tropical cyclone is sensitive to the convective process.

 Sensitivity in model simulation is seen, with MSLP varying by up to 30 hPa and maximum winds by 31 kts among the above four experiments. The observed minimum central MSLP was 962 hPa and the maximum wind was 90 kts. The minimum central MSLP and the maximum surface winds for each convection schemes are calculated and presented in Figures 1(b) and 1(c), respectively. The experiment utilizing the K scheme yields an intensity that is quantitatively much closer to observations than the other forecasts with a minimum MSLP of 967.5 and maximum surface wind of 62 kts. The caveat to these values being that the maximum intensity occurs 30 hours prior to observations. The experiment with B convection scheme predicted a minimum central MSLP of 981 hPa and maximum wind of 52 kts. The experiment with the G convective scheme resulted in a minimum central MSLP of 997.5 hPa and maximum surface winds of 31 kts. Similarly, the experiment with S scheme produced the MSLP of 973 hPa and maximum wind of 52 kts. The time of maximum intensity of the B and S convective schemes nearly matches observations. Although the G convective scheme produced a track most similar to the observed value, its intensity forecast was much worse than the other schemes.

**Figure 2:**
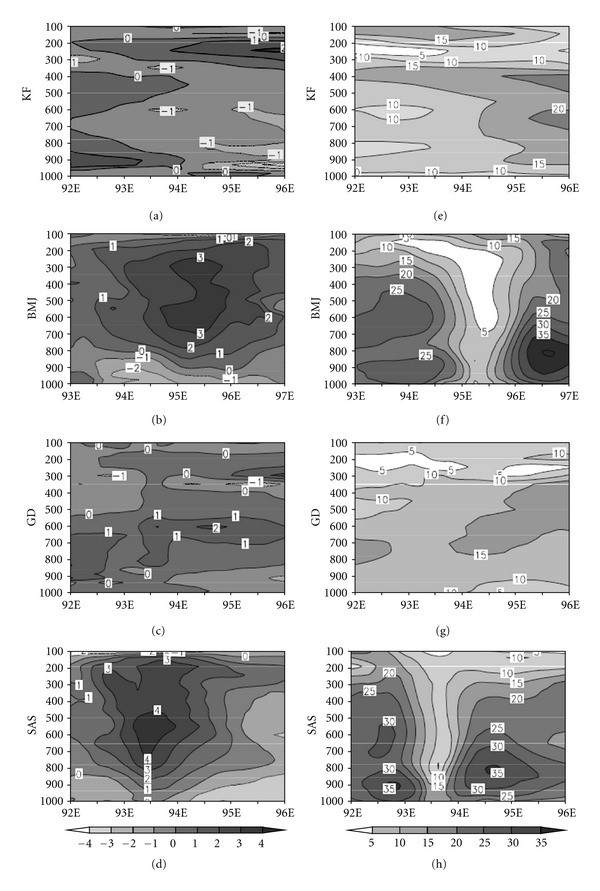
Temperature anomaly (°C) (left panel) and horizontal wind (ms^−1^) (right panel) at the most intense period of the cyclone Nargis with different cumulus convective schemes.

Since cumulus convection schemes play an important role in the development of tropical cyclones, hence to further examine the implication of utilizing different convective schemes, the structure of tropical cyclone is examined.  Figure 2 represents the temperature anomaly and horizontal wind structure at the most intense time of the cyclone. The results clearly suggested that S and B convective schemes are giving similar type of result with a clear representation of the intense structure of the storm; however, G and K convective schemes fail to represent the same. But, at the same time, the K scheme is producing nearly same intensity as observed. Next, the results of PBL sensitivity experiments are presented with K, B, and S convection scheme.

### 5.2. Sensitivity Experiments with PBL Schemes

**Figure 3:**
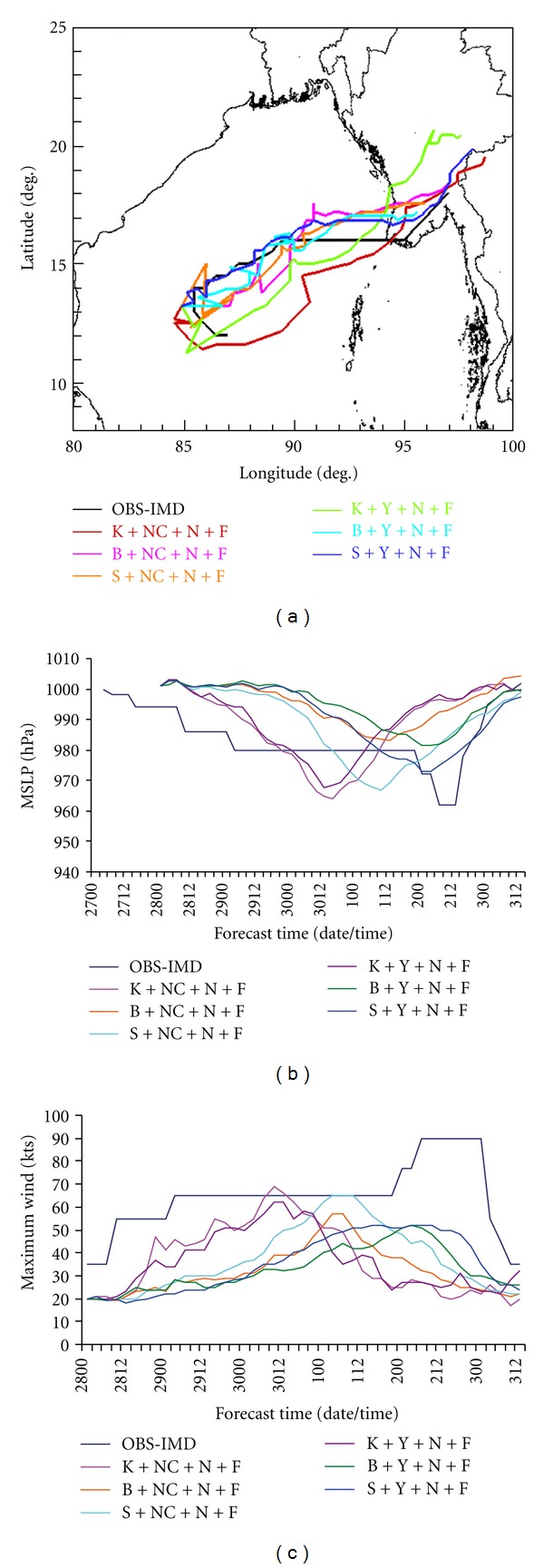
(a) Track, (b) MSLP (hPa), and (c) maximum wind (kts) of the cyclone NARGIS with three best cumulus convections, 2 different PBL, NMM land surface, and Ferrier microphysics option.

 As per the results noted in Section 5.1, the sensitivity of the forecasts to two different PBL schemes YSU (Y) and NCEP GFS (NC) is considered. Hence, another three more experiments are carried out with NC scheme producing a total of 6 experiments for the PBL schemes. The model-simulated track, MSLP, and maximum wind are presented in Figures 3(a), 3(b), and 3(c), respectively. The storm movement is well predicted by the Y scheme than the NC scheme for both B and S convection schemes. However, the K scheme behaves in a different manner with a different PBL scheme. Significant sensitivity is seen in intensity prediction with different PBL schemes. The NC scheme produces a much higher intensity storm than the Y scheme, but it always occur before the observed maximum intensity time. The combination of S + NC + N + F and K + NC + N + F produces the MSLP of 967 hPa and 964 hPa, respectively, whereas S + Y + N + F and K + Y + N + F produces the MSLP of 973 hPa and 968 hPa, respectively. The results indicate that with the NC scheme the MSLPs decreases by nearly 4–6 hPa. This clearly suggested that NC scheme leads to more intense storm, but at an earlier time (nearly 42 hrs for K scheme and 24 hrs for S scheme) than observed. However, the Y scheme simulates intensity reasonably well. Hence, the two best combinations “B + Y + N + F” and “S + Y + N + F” from six combinations are chosen for further investigation.

### 5.3. Sensitivity Experiments with Land Surface Schemes

**Figure 4:**
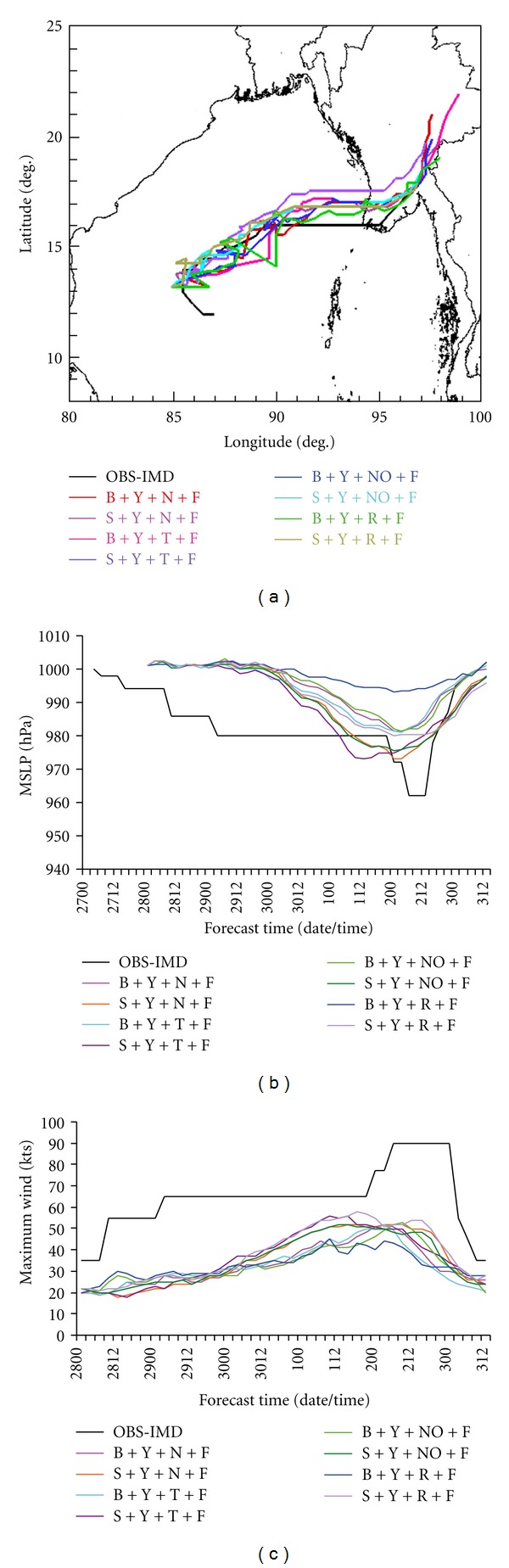
(a) Track, (b) MSLP (hPa), and (c) maximum wind (kts) of the cyclone NARGIS with two best cumulus convections, best PBL, 4 different land surface schemes, and Ferrier microphysics option.

As discussed in the previous subsections, the combination of S and B schemes for convection and Y scheme for PBL produce the better simulation of cyclone Nargis. Hence, another six more experiments are carried out with the available land surface physics schemes to determine the role of surface fluxes of heat and moisture in forecasts of the tropical cyclone. The model-simulated track, MSLP and maximum wind are presented in Figures 4(a), 4(b) and 4(c), respectively along with the IMD observation as part of further analysis of the two best combinations of parameterizations. Track of the cyclone is very well simulated with all the land surface processes. However, significant sensitivity is seen in the intensity prediction and the MSLP varies from 993 hPa to 973 hPa for the various combinations of land surface schemes. It is also noticed that NO and N land surface schemes produce similar results in terms of MSLP and maximum wind in both S and B convection schemes. But the S scheme produces more accurate value towards observation than that of B scheme. Noah (NO) land surface is also producing similar type of result as NMM (N) with both S and B convection schemes in terms of MSLP and maximum wind. But, the S convection scheme is producing more realistic value and comparable to observation value than the B scheme. Hence, the combinations of S + Y + N + F and S + Y + NO + F are selected for further study with microphysics schemes based on their performances.

### 5.4. Sensitivity Experiments with Microphysics Schemes

**Figure 5:**
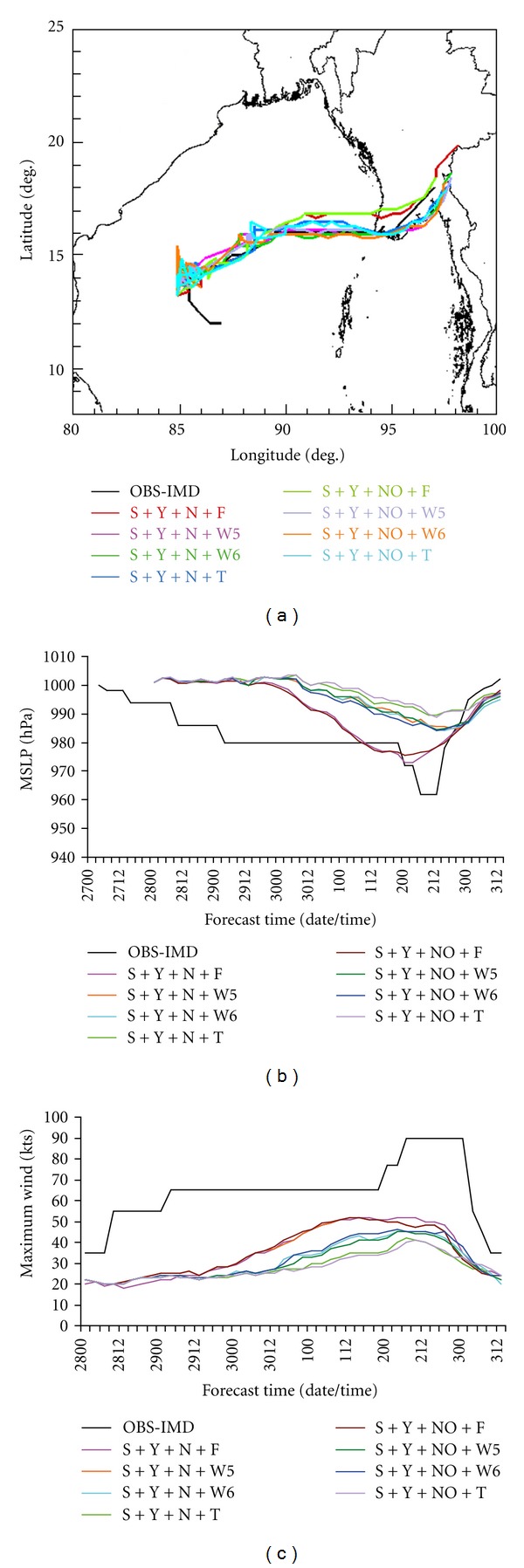
(a) Track, (b) MSLP (hPa), and (c) maximum wind (kts) of the cyclone NARGIS with best cumulus convections, best PBL, 2 best land surface schemes, and 4 different microphysics options.

As per the results noted in previous subsections, the combination of S scheme for convection, Y scheme for PBL, and N and NO schemes for land surface processes produces the better simulation result. Hence, in order to investigate the performance of microphysics schemes, another six experiments are carried out with the different options for microphysics as W5, W6, and T with two experiments each (as we are taking 2 land surface options) make the total eight experiments (two for F) along with S cumulus convection scheme and Y PBL scheme. The model-simulated track, MSLP, and maximum wind are shown in Figures 5(a), 5(b), and 5(c), respectively. The track simulations W5, W6, and T schemes are well matched with observational data as provided by IMD than the F scheme. However, the intensity predictions with W5, W6, and T schemes are very poorly represented. The minimum MSLP as predicted by S + Y + N + F is 973 hPa, whereas it is 985 hPa, 984 hPa, and 990 hPa with S + Y + N + W5, S + Y + N + W6, and S + Y + N + T schemes, respectively. Again, the minimum MSLP as predicted by S + Y + NO + F is 976 hPa, where as it is 985 hPa, 984 hPa, and 989 hPa with S + Y + NO + W5, S + Y + NO + W6, and S + Y + NO + T schemes, respectively. Hence, it may be concluded that the combinations of S + Y + N + F and S + Y + NO + F are producing better result than any other combination. However, it may be noted that the N land surface scheme is giving slightly better result than the NO scheme in terms of movement and intensity of the storm.

### 5.5. Precipitation

**Figure 6:**
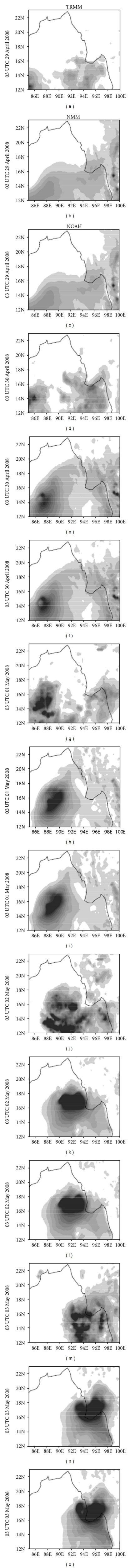
24 hrs accumulated rainfall from TRMM (left panel), NMM land surface (middle panel), and NOAH land surface (right panel) valid at corresponding time.

The results as obtained from the previous subsections clearly show that, the S convection scheme, Y PBL scheme, N and NO land surface schemes, and F microphysics scheme are producing better result than any other combinations.  Figure 6 shows 24 hrs accumulated precipitation as obtained from Tropical Rainfall Measuring Mission (TRMM 3B42) datasets, which is a merger of TMI, other microwave radiometers (SSMI, AQUA), and IR radiometers calibrated using rain gauges and TRMM's precipitation radar and carried out by National Aeronautics and Space Administration (NASA) and model simulations. The precipitation data are obtained from the NASA web site (http://disc2.nascom.nasa.gov/Giovanni/tovas/). The left panel is from TRMM observed precipitation, middle panel is for model simulation with S + Y + N + F combination, and right panel is for S + Y + NO + F combination. The spatial distribution of precipitation is found to be nearly same with both N and NO land surface schemes. However, N scheme is able to produce peak precipitation in terms of both amount and time of occurrence and comparable with observed precipitation than the NO scheme, which has been clearly demonstrated in subsequent section.

### 5.6. Evaluation of Performance of the Model with Different Initial Conditions

As discussed above (Section 4), the model performance is evaluated with the best two combinations after a detailed investigation of different combinations of convection, PBL, land surface, and microphysics schemes. For this purpose, starting from 0000 UTC 28 April 2008 and in every 12 hour interval, the model is integrated up to 0300 UTC 03 May 2008 for each simulation. Thus, another ten experiments (five experiments for each combination) are carried out from the initial condition of 1200 UTC 28 April 2008, 0000 UTC 29 April 2008, 1200 UTC 29 April 2008, 0000 UTC 30 April 2008, and 1200 UTC 30 April 2008.

#### 5.6.1. Simulation of Track and Intensity

**Figure 7:**
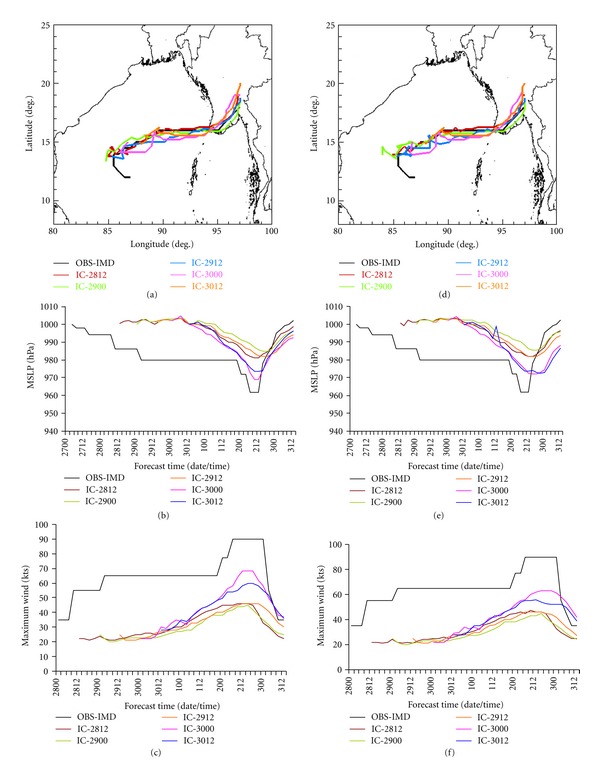
(a) Track, (b) MSLP (hPa), and (c) maximum wind (kts) of the cyclone NARGIS with S cumulus convections, Y PBL, NMM land surface scheme, and Ferrier microphysics option at different initial conditions; (d), (e), and (f) are same as (a), (b), and (c) but with NOAH land surface scheme.

The model simulated track positions, MSLP, and maximum wind along with the IMD observations are presented in Figure 7. Figures 7(a), 7(b), and 7(c) are the results as obtained with S convection, Y PBL, F microphysics, and N land surface which shows that the track and intensity is well simulated by the model. Figures 7(d), 7(e), and 7(f) are the results as obtained with S convection, Y PBL, F microphysics, and NO land surface processes. The track simulations with S + Y + N + F and S + Y + NO + F combinations are providing similar types of results. But, a lot of difference is found in intensity prediction. The S + Y + NO + F combination produces the less intensity prediction and also results in 03 hrs delay in time.

The mean absolute track error (MATE) (km) with the two optimum physics combinations with different initial conditions are evaluated up to 96 hrs of simulation. The mean MATEs are also calculated for the same period. The 24 hrs result shows that there is an improvement of 30.6% with S + Y + N + F combination than S + Y + NO + F options. Similarly, 48, 72, and 96 hrs results clearly show an improvement of 13.5%, 49%, and 30%, respectively, with the S + Y + N + F combination than S + Y + NO + F options. The detailes of the MATEs are presented in Table 2. The landfall point errors (LEs) and landfall time errors are also calculated with the two optimum physics combinations with different initial conditions. Results show that S + Y + N + F combination is giving less landfall point error than the S + Y + NO + F combination, though the landfall time error is same in both the schemes. The detailes of the LEs are presented in Table 3.

#### 5.6.2. Simulation of Precipitation Pattern

**Figure 8:**
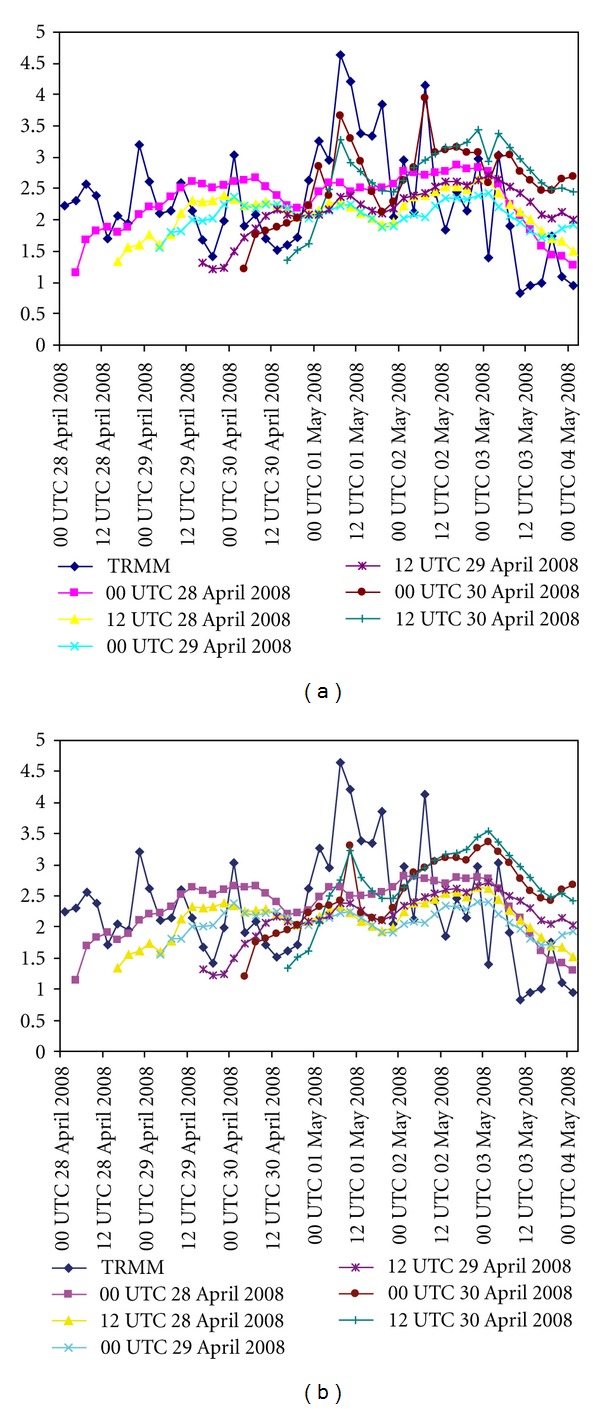
Time-series of area-averaged rainfall (cm hr^−1^) from TRMM and model simulation at different initial conditions. (a) Comparison among TRMM and model simulation with NMM land surface scheme and (b) same as (a) but with NOAH land surface scheme.

 Figures 8(a) and 8(b) demonstrated the time series of area averaged precipitation simulation with S + Y + N + F and S + Y + NO + F combinations, respectively. For the both combinations, the model is integrated at different initial conditions as described above. Also, the time series of area-averaged TRMM precipitation is considered for better comparison. A lot of improvement is seen with N scheme than the NO scheme. Two peak intensities are found from TRMM precipitation at 0600 UTC 01 May 2008 and 0600 UTC 02 May 2008. The model with N scheme is able to simulate the peak precipitation than the NO scheme. At 0600 UTC 01 May 2008, TRMM produced the averaged precipitation of 4.6 mm and model could simulate the precipitation of 3.7 mm and 2.4 mm with N and NO schemes, respectively. Similarly, at 0600 UTC 02 May 2008, TRMM produced averaged precipitation of 4.1 mm and model could simulate the precipitation of 3.9 mm and 2.9 mm with N and NO schemes, respectively. Hence, it may be concluded that the N scheme well-simulates the precipitation than the NO scheme.

#### 5.6.3. Some Characteristic Features of Nargis

**Figure 9:**
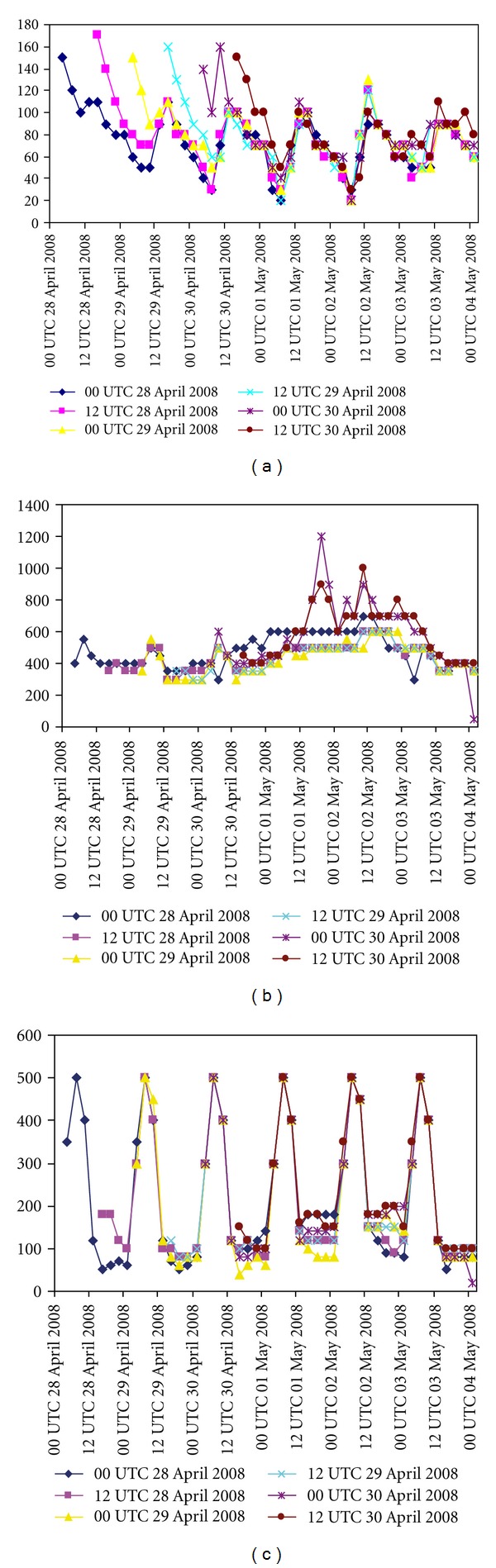
Time series of model-simulated (a) ground heat flux (Wm^−2^), (b) latent heat flux (Wm^−2^), and (c) sensible heat flux (Wm^−2^) at different initial conditions with NMM land surface scheme.

**Figure 10:**
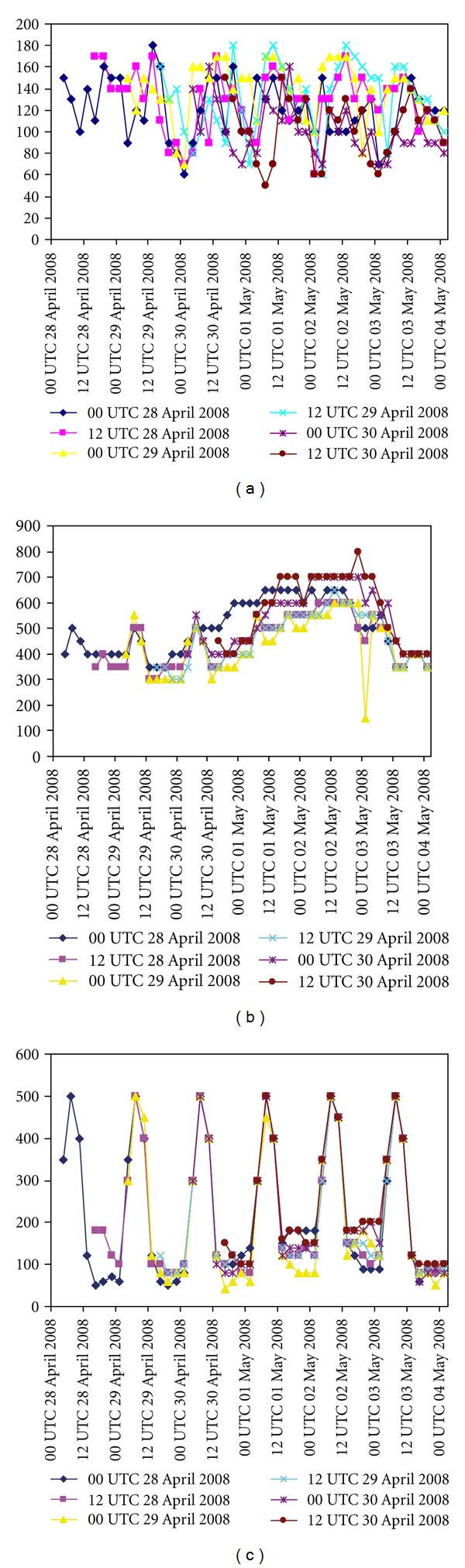
Time series of model simulated (a) ground heat flux (Wm^−2^), (b) latent heat flux (Wm^−2^) and (c) sensible heat flux (Wm^−2^) at different initial conditions with NOAH land surface scheme.

 It has been attempted to study the structure of Nargis in terms of simulation of heat fluxes, vertical velocity, and absolute vorticity at different initial conditions. Figures 9(a), 9(b), and 9(c) represent the model-simulated ground heat fluxes (GHF-), latent heat fluxes (LHF-), and sensible heat fluxes (SHF-) with N scheme and with different initial conditions. Figures 10(a), 10(b), and 10(c) represent the model-simulated ground heat fluxes, latent heat fluxes, and sensible heat fluxes with NO scheme and with different initial conditions. The latent heat flux is one of the dominant components of the air-sea energy exchange processes associated with tropical cyclones. The model simulation with N scheme produced the LHF of 1200 Wm^−2^, whereas the simulation with NO scheme produced the LHF of 800 Wm^−2^.

**Figure 11:**
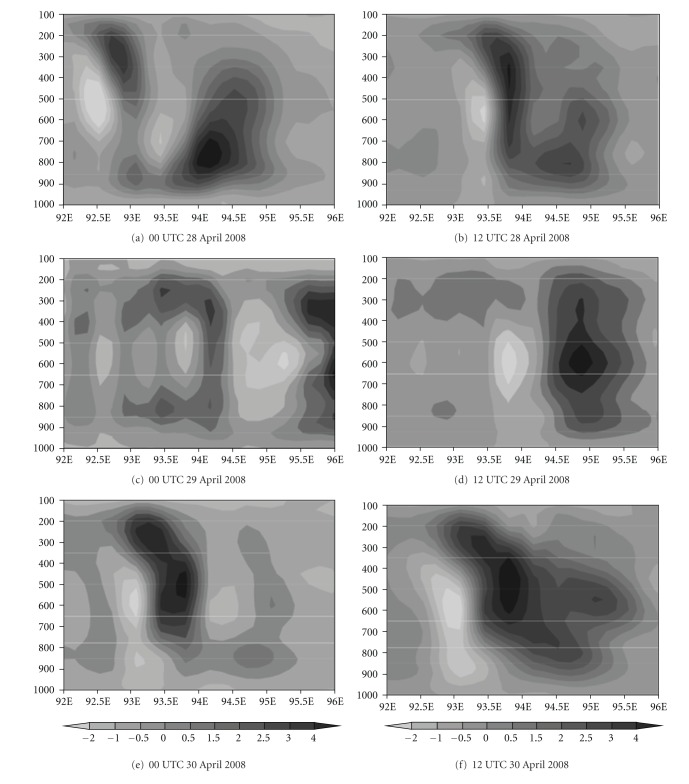
Model-simulated vertical velocity (ms^−1^) at the peak intense time with different initial conditions with NMM land surface scheme.

**Figure 12:**
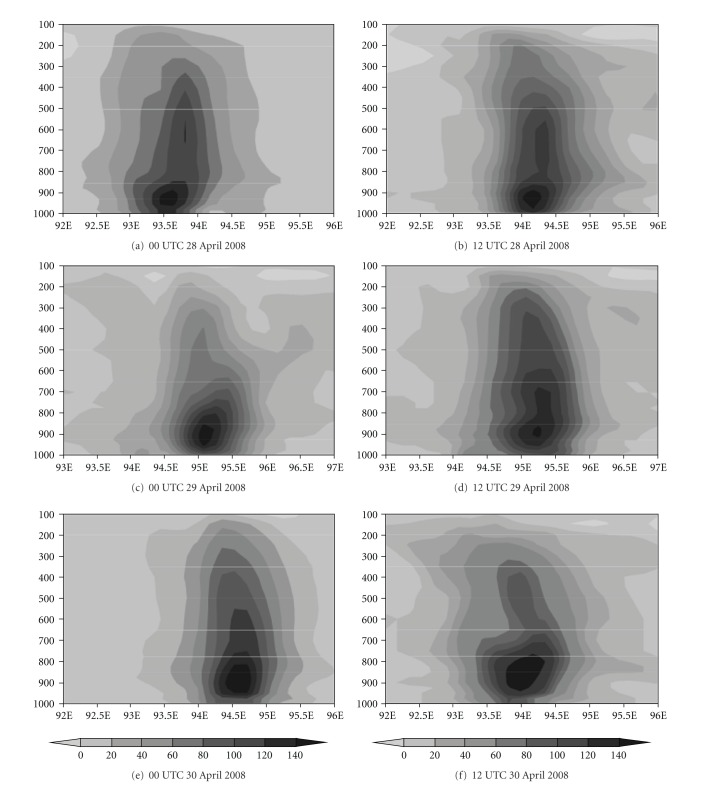
Model-simulated absolute vorticity (×10^−5^ s^−1^) at the peak intense time with different initial conditions with NMM land surface scheme.

 Furthermore, the vertical structure of the storm has been demonstrated with the optimum combination, that is, with S convection, Y planetary boundary layer, N land surface, and F microphysics scheme.  Figure 11 represents the model-simulated vertical velocity at the peak intense time of the system with different initial conditions. The strong updraft and downdraft are noticed from model simulation. The maximum value of 5 ms^−1^ is seen in the middle level and updraft is extended up to 150 hPa.  Figure 12 represents the model-simulated absolute vorticity at the peak intense time with different initial conditions. The positive vorticity of order of 20–140 × 10^−5^  S^−1^ is extended up to 100 hPa. Also, strong positive vorticity is found up to 400 hPa.

All the above results and discussions clearly demonstrate that the S + Y + N + F combination is the optimum combination among all the other combinations in terms of predicting track, intensity, precipitation, and structure of the storm.

## 6. Conclusions

From the present study on the impact of parameterization schemes for simulation of tropical cyclone, the following broad conclusions are drawn.

 The model is sensitive to cumulus convection, planetary boundary layer, and microphysics parameterization schemes. The results from sensitivity experiments with different schemes for cumulus convection indicate that the movement of the cyclone is quite sensitive to the convection processes. The Simplified Arakawa Schubert convection scheme gives better track positions with minimum vector displacement and landfall errors. The result has been clearly demonstrated from the simulation of inner core structure of the storm through temperature anomaly and horizontal wind pattern.

 The results from sensitivity experiments with different PBL schemes indicate that the PBL plays an important role in the intensification of the storm. The NCEP GFS scheme gives early intensification of the storm. However, YSU scheme well-simulated the intensification of the storm which is more comparable with the observed value and intense period of the storm. Also, track is well simulated with YSU scheme.

 The results from different experiments with land surface physics schemes show that the NMM and NOAH land surface schemes are producing similar type of results and performing well than any other schemes. However, the NMM scheme is giving better result in terms of track and intensity prediction of the storm than the NOAH scheme. Similarly, the results from sensitivity experiments with different microphysics schemes show that the Ferrier scheme is providing better result in terms of track and intensity prediction than other schemes considered in this study.

 Further, the results on optimum suitable combination of physical processes in WRF-NMM system are confirmed with additional five different initial values as illustrated in this study. The mean vector displacement error at 24, 48, 72, and 96 hrs are improved by 30%, 13%, 49%, and 30%, respectively, with the optimum combination. The time of occurrence of maximum rainfalls is well captured. Also, the structure of the storm is well predicted with the optimum combination. The results indicate that the combination of Simplified Arakawa Schubert for cumulus convection, Yonsei University planetary boundary layer, NMM land surface, and Ferrier microphysics schemes are providing better result in terms of simulation of track, intensity, and structure of the cyclone than other combinations considered in this study.

## Figures and Tables

**Table 1:** Details of the WRF-NMM model specifications.

Model	NCEP mesoscale model WRF-NMM V3.0.1
Dynamics	Nonhydrostatic with terrain following hybrid pressure sigma vertical coordinate.

Map projection	Rotated lat-lon

Resolution	9 km

No. of vertical levels	51

Horizontal grid scheme	Arakawa E-grid

Time integration scheme	Horizontal: forward-backward scheme
	Vertical: Implicit scheme

Lateral boundary condition	NCEP/NCAR GFS forecast

Radiation scheme	Long wave: GFDL
	Short wave: GFDL

Planetary boundary layer parameterization schemes	(1) NCEP GFS
	(2) Yonsei University (YSU)

Cumulus parameterization schemes	(1) Kain-Fritsch
	(2) Betts-Miller-janjic
	(3) Grell-Devenyi
	(4) Simplified Arakawa Schubert

Land surface physics	(1) NMM
	(2) Thermal diffusion
	(3) Noah
	(4) RUC

Microphysics	(1) Ferrier
	(2) WSM 5-class
	(3) WSM 6-class graupel
	(4) Thompson

**Table 2:** Mean absolute track errors (km) with two optimum physics combinations with different initial conditions.

Initial conditions	Land surface	00 hr	12 hrs	24 hrs	36 hrs	48 hrs	60 hr	72 hrs	84 hrs	96 hrs
1200 UTC 28 Apr 08	NMM	118.68	145.58	22.28	33.97	68.96	57.90	54.00	77.90	144.4
0000 UTC 29 Apr 08		185.75	75.73	55.06	101	10.71	53.44	42.75	53.44	63.8
1200 UTC 29 Apr 08		31.78	19.76	170.6	61.33	106	106.8	15.4	76.8	
0000 UTC 30 Apr 08		59.61	204.7	54.85	88.95	55.5	0.0	0.0		
1200 UTC 30 Apr 08		38.8	77.25	39	69.57	22.23	43.35			

*Mean error*		*86.924*	*104.604*	*68.358*	*70.964*	*52.68*	*52.298*	*28.0375*	*69.38*	*104.1*

1200 UTC 28 Apr 08	NOAH	118.68	192.93	95	73.67	109.65	172.84	64.75	87.76	150.24
0000 UTC 29 Apr 08		185.75	45.37	96.91	56.67	29.62	88.29	47.87	71.24	120.11
1200 UTC 29 Apr 08		31.78	9.29	160.48	81.62	78	88.4	54.46	46.1	
0000 UTC 30 Apr 08		59.61	204.7	54.85	91.5	59.5	0.0	0.0		
1200 UTC 30 Apr 08		38.8	93.43	39	69.57	22.23	43.35			

*Mean error*		*86.924*	*109.144*	*89.248*	*74.606*	*59.8*	*78.576*	*41.77*	*68.367*	*135.175*

*% of improvement*		*0*	*4.34*	*30.6*	*5.1*	*13.5*	*50.3*	*49*	*−1.4*	*30*

**Table 3:** Landfall point errors (km) and landfall time errors (hrs) with two optimum physics combination at different initial conditions.

Initial conditions	Landfall point error (km) with different land surface		Landfall time error (hrs) with different land surface	
	NMM	NOAH	NMM	NOAH
0000 UTC 28 Apr 08	92.4	123.3	−9	−9
1200 UTC 28 Apr 08	54.8	64.2	−6	−6
0000 UTC 29 Apr 08	7.8	7.8	−6	−6
1200 UTC 29 Apr 08	32	34.3	−6	−6
0000 UTC 30 Apr 08	53.4	54.8	0	0
1200 UTC 30 Apr 08	53.4	75.8	0	0
